# Analytical methodologies based on LC–MS/MS for monitoring selected emerging compounds in liquid and solid phases of the sewage sludge

**DOI:** 10.1016/j.mex.2016.04.010

**Published:** 2016-04-27

**Authors:** C. Boix, M. Ibáñez, D. Fabregat-Safont, E. Morales, L. Pastor, J.V. Sancho, J.E. Sánchez-Ramírez, F. Hernández

**Affiliations:** a Research Institute for Pesticides and Water, University Jaume I, Avda. Sos Baynat, E-12071 Castellón, Spain; b Depuración de Aguas del Mediterráneo, Avda. Benjamin Franklin 21 Parque Tecnológico, Paterna, Spain

**Keywords:** Quantification of selected emerging compounds in liquid and solid phases of the sewage sludge by LC–MS/MS, Emerging contaminants, sewage sludge, solid-phase extraction, ultrasonic-assisted extraction, liquid chromatography, tandem mass spectrometry

## Abstract

In this work, two analytical methodologies based on liquid chromatography coupled to tandem mass spectrometry (LC–MS/MS) were developed for quantification of emerging pollutants identified in sewage sludge after a previous wide-scope screening. The target list included 13 emerging contaminants (EC): thiabendazole, acesulfame, fenofibric acid, valsartan, irbesartan, salicylic acid, diclofenac, carbamazepine, 4-aminoantipyrine (4-AA), 4-acetyl aminoantipyrine (4-AAA), 4-formyl aminoantipyrine (4-FAA), venlafaxine and benzoylecgonine. The aqueous and solid phases of the sewage sludge were analyzed making use of Solid-Phase Extraction (SPE) and UltraSonic Extraction (USE) for sample treatment, respectively. The methods were validated at three concentration levels: 0.2, 2 and 20 μg L^−1^ for the aqueous phase, and 50, 500 and 2000 μg kg^−1^ for the solid phase of the sludge. In general, the method was satisfactorily validated, showing good recoveries (70–120%) and precision (RSD < 20%). Regarding the limit of quantification (LOQ), it was below 0.1 μg L^−1^ in the aqueous phase and below 50 μg kg^−1^ in the solid phase for the majority of the analytes. The method applicability was tested by analysis of samples from a wider study on degradation of emerging pollutants in sewage sludge under anaerobic digestion.

The key benefits of these methodologies are:

• SPE and USE are appropriate sample procedures to extract selected emerging contaminants from the aqueous phase of the sewage sludge and the solid residue.

• LC–MS/MS is highly suitable for determining emerging contaminants in both sludge phases.

• Up to our knowledge, the main metabolites of dipyrone had not been studied before in sewage sludge.

## Graphical abstract

**Figure:**
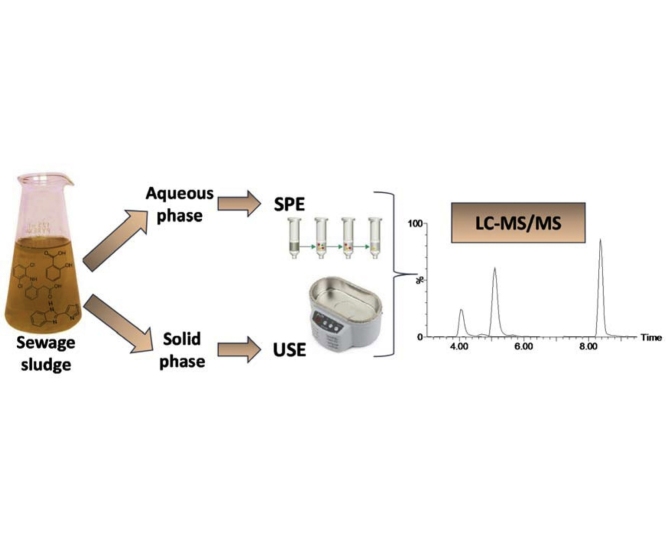

## Method details

The methods developed in this work were applied to investigate the presence of selected emerging contaminants in the aqueous and solid phases of sewage sludge. Both phases were analyzed individually using an optimized extraction procedure for each one. The samples were collected after anaerobic digestion performed in an experimental sewage sludge treatment plant located in the Mediterranean area, using two different types of anaerobic bacteria [1].

The analytes selected for this work were previously found in the sewage samples in a wide-scope screening using UHPLC-QTOF MS [1]. Based on screening data, 13 emerging pollutants were selected to perform quantitative analysis by LC–MS/MS: the fungicide thiabendazole (widely used as post-harvest fungicide in citrus), and the pharmaceuticals carbamazepine (used as a treatment of epilepsy), venlafaxine (anti-depressant), irbesartan and valsartan (used to treat high blood pressure) and the non-steroidal anti-inflammatory diclofenac. Moreover, some metabolites were also selected, such as salicylic acid (which is the main metabolite of acetylsalicylic acid [2], [3]) and three metabolites of the analgesic dipyrone [3], [4], [5] (4-aminoantipyrine (4-AA), 4-acetyl aminoantipyrine (4-AAA) and 4-formyl aminoantipyrine (4-FAA)). Benzoylecgonine (BE), the main metabolite of cocaine, was also considered as this compound, found in the screening of samples at high abundance, is commonly present at high concentrations in urban wastewaters [6], [7].

## Sludge samples origin

The samples were collected from October 2014 to January 2015 in a pilot sludge digestion plant located in the Mediterranean area. Influent sewage sludge samples were collected as 24-h composite of the feeding tank, and grab samples were collected after anaerobic digestion. Samples were frozen at −24 °C immediately after collection. The analyte concentrations were obtained in the aqueous and solid phase of the sludge, in order to estimate the pollutant distribution in both phases [1].

## Sample treatment

Sewage sludge samples contained approximately 2% (w/w) of suspended solids (solid phase). This percentage varied slightly depending on the sample. Before analysis, raw sludge samples were thawed at room temperature, and the aqueous and solid phases separated by decantation. The aqueous phase was centrifuged and the supernatant was subjected to SPE for pre-concentration, clean-up and extraction [8], [9], [10], [11]. In relation to the solid phase, it was lyophilized under vacuum in order to eliminate the residual water and preserve its integrity. An ultrasonic assisted extraction (USE) procedure [12], [13], [14], [15], [16], was then applied for extraction of analytes in the lyophilized solid residue of the sludge.

### Aqueous phase of the sludge

Water samples were centrifuged at 4600 rpm for 10 min in falcon tubes using a Consul21 centrifuge (Ortoalresa, Madrid, Spain). 50 mL of supernatant was spiked at 0.1 μg L^−1^ with the mix of ILIS compounds measured in ESI+, and at 0.4 μg L^−1^ with salicylic acid-d_4_ which is monitored in ESI-.

SPE was performed using Oasis HLB 200 mg cartridges, previously conditioned with 6 mL MeOH and 6 mL Milli-Q water. Samples were two-fold diluted with Milli-Q water and percolated through the cartridges by gravity. After drying under vacuum, the analytes were eluted with 10 mL MeOH. The extracts were evaporated to dryness under vacuum at 60 °C using a miVac DUO concentrator (GeneVac, Suffolk, UK) and finally reconstituted with 1 mL MeOH:water (10:90, v/v). The final extracts were centrifuged in Eppendorf tubes at 12000 rpm for 10 min (Centronic-BL, Selecta, Barcelona, Spain), and the supernatants were transferred into LC vials, and injected in the LC–MS system. (For further details about SPE optimization see SI).

### Solid phase of the sludge

Lyophilized solid phase was homogenized using a glass mortar. 0.1 g of homogenized solid residue were accurately weighted (precision 0.1 mg) directly in Eppendorf tubes and spiked at 0.05 mg kg^−1^ with the mix of ILIS compounds measured in ESI+, and at 0.2 mg kg^−1^ with salicylic acid-d_4_ which is monitored in ESI-. Afterwards, it was extracted with 2 mL of MeOH:water (50:50) 0.5% HCOOH, using a Vortex (Heidolph Instruments, Schabach, Germany) for 1 min. After that, samples were sonicated 15 min at 40 kHz (Selecta Ultrasons, Selecta, Barcelona, Spain) and centrifuged at 12000 rpm for 10 min. The supernatant was collected into a glass tube, and the extraction procedure was repeated. Both supernatants were mixed and evaporated to dryness under vacuum at 60 °C using a miVac DUO concentrator, and finally reconstituted with 1 mL of MeOH:water (10:90, v/v). The final extract was centrifuged at 12000 rpm for 10 min and transferred into a LC vial for its injection in the LC–MS. (For further details about USE optimization see SI).

## Materials and instrumentation

The list of the target compounds for instrumental method development is presented in Table 1. Reference standards were purchased from Toronto Research Chemicals (Ontario, Canada), Sigma-Aldrich (St Louis, MO, USA), LGC Promochem (London, UK), Across Organics (Geel, Belgium), Bayer Hispania (Barcelona, Spain), Fort Dodge Veterinaria (Gerona, Spain), Aventis Pharma (Madrid, Spain) and Vetoquinol Industrial (Madrid, Spain). Benzoylecgonine (BE) was obtained from Cerilliant (Round Rock, TX, USA).

Regarding Isotopically-Labeled Internal Standards (ILIS), benzoylecgonine-d_3_ was purchased from Cerilliant as solution in methanol at a concentration of 100 mg L^−1^. The rest of the ILIS (Table 1) were purchased from CDN Isotopes (Quebec, Canada).

Formic acid (HCOOH, content > 98%), HPLC-grade methanol (MeOH), HPLC-grade acetonitrile (ACN), sodium hydroxide (NaOH, >99%) and ammonium acetate (NH_4_Ac) were acquired from Scharlab (Barcelona, Spain). HPLC-grade water was obtained from deionized water passed through a Milli-Q Gradient A10 (18.2 MΩ cm) water purification system (Millipore, Bedford, MA, USA).

Solid-phase extraction (SPE) cartridges (Oasis HLB; 200 mg) were purchased from Waters (Milford, MA, USA).

The analytical instrumentation consisted of a Waters Alliance HT 2795 high performance liquid chromatography (HPLC) system (Waters, Mildford, MA, USA), equipped with a quaternary solvent manager and a sample manager coupled to a triple quadrupole mass spectrometer Quattro Micro API MS (Waters) equipped with an orthogonal Z-spray electrospray ionization interface (ESI) operated in both positive and negative ion modes.

Cone gas as well as desolvation gas was nitrogen (Praxair, Valencia, Spain) set up 60 L h^−1^ and 600 L h^−1^, respectively. For operation in the MS/MS mode, collision gas was argon with 99.995% purity (Praxair, Valencia, Spain). Other parameters optimized were capillary voltage 3.5 kV (ESI + ) and −3 kV (ESI-), source temperature 120 °C and desolvation temperature 350 °C. The cone voltage was optimized for each compound. Dwell times of 0.1 s were selected as a compromise between sensitivity and number of points per peak.

Chromatographic separation was performed using a Symmetry C18 analytical column (50 × 2.1 mm, 3.5 μm particle size, Waters). A gradient consisting of water (solvent A) and MeOH (solvent B) both with 0.1 mM NH_4_Ac and 0.01% HCOOH was used as mobile phase. The percentage of MeOH was changed linearly as follows: 0 min, 10%; 6 min, 90%; 7 min, 90%; 7.1 min, 10%. The total run time was 12 min. The flow rate was 300 μL min^−1^. The sample injection volume was 20 μL.

All data were acquired and processed using MassLynx version 4.1 (Waters). The quantification process was performed using TargetLynx application manager (Micromass v4.1).

## LC–MS optimization

In this work, different mobile phases (water, MeOH and acetonitrile) with different additives (HCOOH and NH_4_Ac) were tested. The effect of pH and ionic strength of mobile phase on peak shape, resolution and peak area were evaluated by varying the modifier concentration.

MS/MS parameters such as Selected Reaction Monitoring (SRM) transitions, cone voltage and collision energy as well as retention time are listed in Table 1. For the MS/MS optimization, individual standard solutions were directly infused in the triple quadrupole (QqQ). Most of the compounds (10 out of 13) were determined in ESI operating in positive ionization mode, using the protonated molecule [M + H]^+^ as precursor ion. The 3 remaining compounds were determined in negative ionization mode, using the deprotonated molecule [M-H]^−^ as precursor ion. The two most intense SRM transitions (in terms of peak area) were selected for each compound: the most abundant product ion was used for quantification (Q) and one additional product ion was used for confirmation (q) (Table 1). Regarding ILISs, only the most intense transition was monitored.

## Matrix effects

Severe matrix effects were observed due to the complexity of the samples, mostly leading to ionization suppression. In order to avoid complicated sample treatment with clean-up steps, we used ILIS for correction of the signal suppression ensuring an accurate quantification [9], [17]. In addition, using ILIS as surrogate, potential analyte losses during sample treatment could be also corrected. In total, 7 ILIS (thiabendazole-d_6_, venlafaxine-d_6_, BE-d_3_, irbesartan-d_6_, valsartan-d_8_, diclofenac-d_4_ and salicylic acid-d_4_) were used for quantification of their corresponding analytes, while 4 more compounds were quantified using analogue ILIS, BE-d_3_ for the quantification of 4-AA, 4-FAA and 4-AAA and irbesartan-d_6_ for carbamazepine. In this case, the selection of the analogue ILIS was mainly based on retention time similarity between analyte and ILIS, in order to facilitate that both compounds were affected by similar constituents of the matrix. The remaining 2 compounds, acesulfame and fenofibric acid, could not be properly corrected by any ILIS available in our laboratory.

## Method validation

The two methodologies developed for the determination of the 13 selected emerging compounds in the aqueous and solid phase of sewage sludge were finally validated.

The linearity of the method was studied by injecting different concentration levels in duplicate in the range 0.05–500 μg L^−1^, establishing the adequate lineal range for each compound (see Table 2). Calibration curves showed, in all cases, correlation coefficients (r) greater than 0.99, based on relative responses (analyte peak area/ILIS peak area), and residuals lower than 10%.

**Fig. 1:**
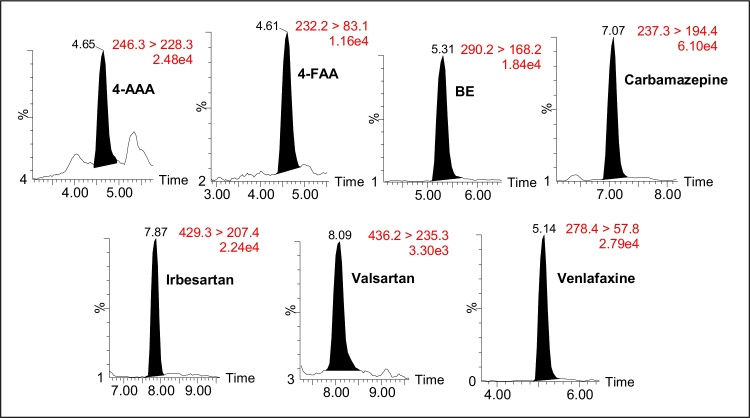
LC–MS/MS chromatograms for compounds at the lowest level validated (50 μg kg^−1^) in the solid phase of the sludge.

Method accuracy (estimated by means of recovery experiments in spiked samples) and precision (expressed as repeatability, in terms of Relative Standard Deviation (RSD)) were evaluated in samples spiked at three concentration levels: 0.2, 2 and 20 μg L^−1^ for the aqueous phase, and 50, 500 and 2000 μg kg^−1^ for the solid phase. The concentration levels tested in this work were selected based on those estimated from the previous LC-QTOF screening performed in the samples. These values were in the line of sewage sludge concentrations reported in most of the published papers, commonly from 40 to 25000 μg kg^−1^ [12], [15], [18], [19]. Both sludge phases were spiked before the sample treatment. The experiments were performed in triplicates (n = 3) for each matrix and each spiking level. As almost all the non-spiked samples used in the validation study contained the target analytes, the concentrations found in these samples were subtracted from those found in spiked samples. Satisfactory recoveries values between 70% and 120%, with RSD lower than 20% were obtained for all contaminants, with some exceptions (see Table 2**)**. For the 4-AA, recovery values lower than 40% were obtained in both sludge phases at all the spiking levels tested. These poor recoveries could be explained as the use of an analogue ILIS (BE-d_3_) could not completely compensate for the matrix effect obtained. Some compounds (4-AAA, 4-FAA, carbamazepine, salicylic acid, thiabendazole, valsartan and venlafaxine) presented RSD higher than 20%, for at least one spiking level. Despite of that, all of these values were lower than 25%, except for thiabendazole in the aqueous phase (42%). It could be originated by the high concentration of this analyte in the samples used as “blank”, which could increase the RDS of the replicates of spiked samples. At the lowest level, several compounds in both aqueous phase and solid phase could not be validated, due to the high analyte concentration found in the non-spiked samples. Fig. 1 shows LC–MS/MS chromatograms (quantification transition) for the seven compounds that could be validated at the lowest level in the solid phase of the sewage sludge.

The limit of quantification (LOQ) was estimated for a signal to noise (S/N) ratio 10 from the sample chromatograms at the lowest validation level tested (n = 3), using the quantification transition. As experiments were performed in real-world sewage sludge, no blank samples were available for most of analytes. In those cases, LOQ values were estimated from the chromatograms of the non-spiked samples taking into account the concentration levels found in them. As can be seen in Table 2, most LOQs were below 0.1 μg L^−1^ and 50 μg kg^−1^ in the aqueous and solid phases, respectively.

The calorie-free sweetener, acesulfame, and the main metabolite of the fenofibrate, fenofibric acid, could not be properly corrected by any ILIS used; as a consequence, and due to the strong matrix effects observed, they could not be satisfactory validated. For these compounds, absolute response of samples were used to roughly evaluate trends in their possible degradation after sewage sludge treatment [1].

## Confirmation of the identity

Confirmation of the identity of compounds in samples was based on the agreement in LC retention time (±0.2 min) and the “q/Q” intensity ratio (±30%) in relation to the reference standard [20].

**Fig. 2:**
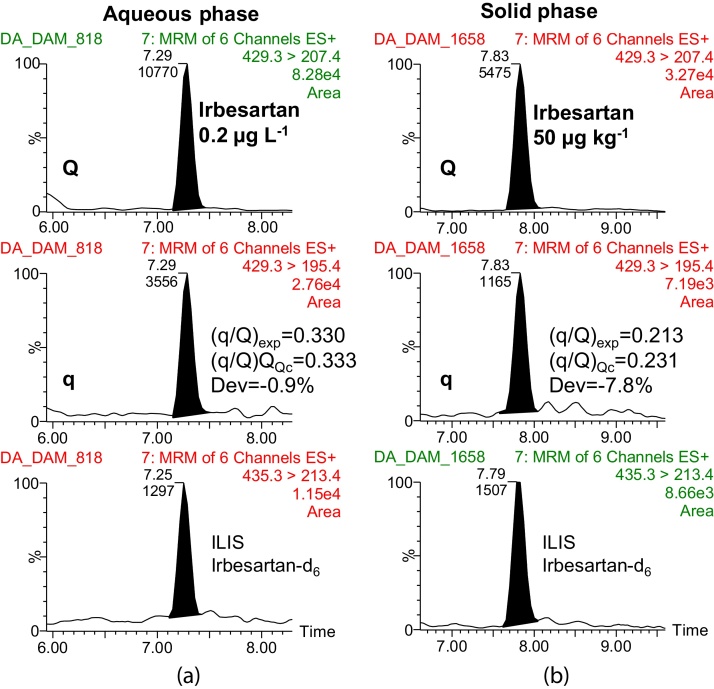
LC–MS/MS chromatograms for compounds at the lowest level validated (50 μg kg^−1^) in the solid phase of the sludge.

As an example, Fig. 2 shows the detection and confirmation of the pharmaceutical irbesartan in the (a) aqueous and (b) solid phases of the sludge at the lowest level validated, 0.2 μg L^−1^ and 50 μg kg^−1^, respectively. As it can be seen, “q/Q” ratio was within the established limits, being, in this case, lower than 10%.

## Analysis of sewage sludge samples

A total of 50 sewage sludge samples, which resulted in 50 aqueous phase and 50 solid phase samples, collected from four anaerobic experiments under thermophilic or mesophilic bacteria, were analyzed using the analytical methodology developed and validated in this work. The objective was to evaluate degradation efficiency and distribution of the 13 emerging pollutants in the two phases of sewage sludge. Quality controls for both matrices were included in every sequence, presenting satisfactory recoveries (in the range of 70–120%) for the majority of the analytes. The compounds under study were convincingly identified and quantified at concentrations higher than the LOQs. The results of that work are reported and discussed in [1].

## Figures and Tables

**Table 1:** MS/MS optimized conditions for selected compounds.

Compound	ESI	RT (min)	Cone (V)	Q transition	CE (eV)	q transition	CE (eV)
4-AA	+	3.91	20	204.4 > 83.1	15	204.4 > 159.1	10
4-AAA	+	4.58	20	246.3 > 228.3	20	246.3 > 83.1	20
4-FAA	+	4.55	20	232.2 > 83.1	20	232.2 > 104.1	20
Acesulfame	–	6.32	20	162.2 > 82.0	15	162.2 > 77.9	30
BE	+	5.26	30	290.2 > 168.2	20	290.2 > 105.1	30
Carbamazepine	+	7.13	30	237.3 > 194.4	20	237.3 > 192.4	45
Diclofenac	+	8.51	20	296.2 > 214.2	30	296.2 > 278.1	10
Fenofibric Acid	–	8.71	20	317.2 > 231.2	20	317.2 > 195.3	35
Irbesartan	+	7.98	30	429.3 > 207.4	25	429.3 > 195.4	20
Salicylic Acid	–	7.50	20	137.1 > 93.1	15	137.1 > 65.0	25
Thiabendazole	+	4.80	40	202.3 > 175.2	25	202.3 > 131.2	30
Valsartan	+	8.21	20	436.2 > 235.3	20	436.2 > 207.2	25
Venlafaxine	+	5.08	20	278.4 > 57.8	15	278.4 > 260.4	10
BE-d_3_	+	5.26	30	293.3 > 171.2	20		
Diclofenac-d_4_	+	8.51	20	300.1 > 219.2	20		
Irbesartan-d_6_	+	7.98	30	435.3 > 213.4	25		
Salicylic Acid-d_4_	–	7.50	20	141.1 > 97.1	15		
Thiabendazole-d_6_	+	4.80	40	208.3 > 136.0	30		
Valsartan-d_8_	+	8.21	20	444.3 > 291.2	20		
Venlafaxine-d_6_	+	5.08	20	284.3 > 63.9	15		

ESI: electrospray ionization; RT: retention time; Q: quantification transition; q: confirmation transition; CE: collision energy.

**Table 2:** Results of the validation for aqueous and solid phases of sewage sludge. Limit of quantification (LOQ), recovery (%) and relative standard deviation (RSD) at the three validation levels tested.

Compound	Aqueous Phase (n = 3)				Solid Phase (n = 3)				ILIS (Surrogate)	r (lineal range, μg L^−1^)	
	% Recovery (RSD)			LOQ (ng L^−1^)	% Recovery (RSD)			LOQ (μg kg^−1^)			
	0.2 μg L^−1^	2 μg L^−1^	20 μg L^−1^		50 μg kg^−1^	500 μg kg^−1^	2000 μg kg^−1^				
4-AA	n.v.	37 (19)	27 (9)	99	n.v.	23 (23)^a^	20 (21)	11	BE-d_3_	0.993	(0.5–100)
4-AAA	n.v.	106 (20)	93 (6)	33	76 (4)^a^	110 (24)^a^	92 (21)	46	BE-d_3_	0.999	(0.1–50)
4-FAA	n.v.	101 (16)	99 (5)	96	97 (16)	105 (23)^a^	111 (14)	46	BE-d_3_	0.999	(0.1–50)
BE	103 (10)	96 (3)	94 (2)	9.3	88 (10)	86 (15)	90 (8)	15	BE-d_3_	0.999	(0.05−500)
Carbamazepine	88 (23)	92 (21)	116 (8)	8.6	114 (11)	114 (13)	117 (14)	5.4	Irbesartan-d_6_	0.999	(0.05−500)
Diclofenac	n.v.	96 (1)^a^	99 (24)^a^	48	n.v.	114 (15)	98 (7)	63	Diclofenac-d_4_	0.999	(0.05−500)
Irbesartan	107 (7)	98 (3)	103 (9)	14	80 (12)	90 (9)	97 (14)	1.2	Irbesartan-d_6_	0.999	(0.1–500)
Salicylic Acid	n.v.	n.v.	95 (22)	220	n.v.	n.v.	109 (17)	35	Salicylic Acid-d_4_	0.995	(0.05−500)
Thiabendazole	n.v.	n.v.	114 (42)	84	n.v.	122 (1)^a^	92 (11)	17	Thiabendazole-d_6_	0.999	(0.5–500)
Valsartan	n.v.	105 (6)^a^	100 (4)	74	66 (8)^a^	76 (22)	100 (20)	27	Valsartan-d_8_	0.998	(0.25–500)
Venlafaxine	98 (21)	96 (2)	107 (6)	0.2	82 (24)	91 (15)	92 (19)	7.5	Venlafaxine-d_6_	0.999	(0.1–500)

n.v.: These compounds could not be validated as all the three samples used as “blank” contained high analyte concentration.

a Validation performed for n = 2, due to the high analyte concentration found in one of the three “blank” samples used for validation.
