# Supplementary material for: Analytical methodologies based on LC–MS/MS for monitoring selected emerging compounds in liquid and solid phases of the sewage sludge
Source: MethodsX. 2016 Apr 27;3:333–42. doi: 10.1016/j.mex.2016.04.010 (PMC4865632; doi:10.1016/j.mex.2016.04.010)
Supplement: Supplementary file 1 [file mmc1.docx]

**Supplementary Information**

**Analytical methodologies based on LC-MS/MS for monitoring selected emerging compounds in liquid and solid phases of the sewage sludge**

**C. Boix^a^, M. Ibáñez^a^, D. Fabregat-Safont^a^, E. Morales^b^, L. Pastor^b^, J.V. Sancho^a^, J.E. Sánchez-Ramírez^b^, F. Hernández^a^***

^a^ Research Institute for Pesticides and Water, University Jaume I, Avda. Sos Baynat, E-12071, Castellón, Spain

^b^ Depuración de Aguas del Mediterráneo, Avda. Benjamin Franklin 21 Parque Tecnológico, Paterna, Spain

** Corresponding author* [felix.hernandez@uji.es](mailto:felix.hernandez@uji.es)*, Tel +34 964 387366, Fax +34 964 387368*

**1. Sample treatment optimization**

**1.1. SPE optimization**

Oasis HLB 60 mg and Oasis HLB 200 mg cartridges were tested in order to optimize the SPE procedure. 60 mg cartridges were not appropriate as they got blocked by the matrix leading to a small breakthrough volume. Consequently, Oasis HLB 200 mg cartridges were selected.

In order to stablish the appropriate sample volume of the aqueous sludge phase, four spiked samples were prepared: (A) 50 mL of sludge aqueous phase, (B) 50 mL of sludge aqueous phase diluted to a final volume of 100 mL with Milli-Q water (this is, 1:1 dilution), (C) 20 mL of aqueous phase diluted until a final volume of 100 mL using Milli-Q water (1:5 dilution), and (D) 1:5 dilution filtered using a mixed cellulose ester membrane filter 0.45 µm (Whatman). The four samples were passed through the Oasis cartridges. Sample A blocked the cartridge due to the complex matrix and sample D blocked the cellulose filter. On the contrary, samples B and C passed easily through the cartridge without using vacuum. Sample B was selected in order to maximize the sensitivity of the procedure. Methanol was used as elution solvent to extract analytes from the cartridges [1]

**1.2. USE optimization**

Three different organic solvents mixtures were tested in the lyophilized sewage sludge, using an ultrasonic bath to assist the extraction: MeOH:acetone (1:1) [2], MeOH:dichloromethane (1:1) [3] and MeOH:water (1:1) + 0.5% HCOOH [4]. The extraction procedures which used non-polar solvents (acetone and dichloromethane) showed an incomplete re-dissolution of the extract by the initial mobile phase mixture. For this reason, the last extraction procedure tested was selected.

**References**

[1] R. Díaz, M. Ibáñez, J.V. Sancho, F. Hernández, Qualitative validation of a liquid chromatography-quadrupole-time of flight mass spectrometry screening method for organic pollutants in waters, J. Chromatogr. A. 1276 (2013) 47–57. doi:10.1016/j.chroma.2012.12.030.

[2] T.A. Ternes, M. Bonerz, N. Herrmann, D. Löffler, E. Keller, B.B. Lacida, et al., Determination of pharmaceuticals, iodinated contrast media and musk fragrances in sludge by LC tandem MS and GC/MS, J. Chromatogr. A. 1067 (2005) 213–223. doi:10.1016/j.chroma.2004.10.096.

[3] E. Topuz, S. Sari, G. Ozdemir, E. Aydin, E. Pehlivanoglu-Mantas, D. Okutman Tas, Optimization of diclofenac quantification from wastewater treatment plant sludge by ultrasonication assisted extraction., J. Chromatogr. B. Analyt. Technol. Biomed. Life Sci. 958 (2014) 48–54. doi:10.1016/j.jchromb.2014.02.047.

[4] P. Gago-Ferrero, V. Borova, M.E. Dasenaki, Ν.S. Τhomaidis, Simultaneous determination of 148 pharmaceuticals and illicit drugs in sewage sludge based on ultrasound-assisted extraction and liquid chromatography-tandem mass spectrometry., Anal. Bioanal. Chem. 407 (2015) 4287–97. doi:10.1007/s00216-015-8540-6.
